# Herpes Simplex Virus Type 2 Seroprevalence in Pregnant Women in Urmia, Northwest of Iran, during 2014-2015

**DOI:** 10.29252/ibj.24.2.136

**Published:** 2019-11-01

**Authors:** Zakieh Rostamzadeh Khameneh, Nariman Sepehrvand, Mahshid Mohammadian

**Affiliations:** 1 Solid Tumor Research Center, Urmia University of Medical Sciences, Urmia, Iran;; 2 Research Fellow and Graduate Student, Mazankowski Alberta Heart Institute, University of Alberta, Canada;; 3 Department of Medical Laboratory Sciences, School of Allied Medical Sciences, Urmia University of Medical Sciences, Urmia, Iran

**Keywords:** Herpes simplex virus type 2, Sexually transmitted disease, Iran

## Abstract

**Background:**

HSV-2 seroprevalence has been shown to be a potential sign of infection in pregnant women, and it could be applied to check HSV-2 transmission. This study evaluated the anti-HSV-2 IgG prevalence in pregnant women who were referred to health centers in Urmia, Northwest of Iran, during 2014-2015.

**Methods:**

Serum samples were collected from 86 pregnant women and tested for Anti-HSV-2-specific IgG using a commercial enzyme-linked immunosorbent assays kit.

**Results:**

Five (5.8%) pregnant women showed the presence of Anti-HSV-2-specific IgG antibodies. Previous abortion was reported in 16 (19.7%) and 2 subjects in the seronegative and seropositive groups, respectively.

**Conclusion:**

Data from the present study indicate a lower number of HSV-2 seropositives among the pregnant women in Urmia. This reduction would be a result of low number of studied subjects used in the present study; hence, assessing a large sample is recommended.

## INTRODUCTION

HSV-2 is a double-stranded DNA virus from the human herpes that belongs to Herpesviridae family. This virus is the main source of genital herpes^[^^1^^]^ that attaches to human epidermal and mucosal cells and transfers the enveloped virions to the neuronal cells, then remains in a latent phase^[^^2^^]^. Previous data have indicated the possible relationships between the HSV-2 infection and development of HIV infection and cervical cancer (especially the squamous cell carcinoma)^[^^3^^-^^8^^]^. Globally, there is a geographic variation in the contagion of this specific sexually transmitted infection^[^^5^^,^^9^^]^. Prevalence of HSV-2 among women can vary according to the area, which varies from 17% in USA to 80% in Sub-Saharan Africa. Overall, HSV-2 infection rates are higher among women than men and among pregnant than non-pregnant women^[^^9^^,^^10^^]^.

Genital herpes is a common sexually transmitted infection in the world^[^^11^^]^, and most infected people are not aware of their infection. Hence, they expand and transfer the infection to healthy individuals, even those with no clinical symptoms^[^^12^^]^. Most cases of HSV-2-seropositive patients are asymptomatic and unaware from their genital herpes infection^[^^13^^]^; therefore, the high rate of individuals with HSV-2 infection remains as long-lasting carriers. 

Based on previous data, the HSV-2 seroprevalence was 15.7% among 14-49-year-old individuals in the United States^[^^14^^]^. In addition, a previous study has reported that 25% of infected individuals (with earliest symptomatic episode of this disease) have antibodies against HSV-2. Indeed, HSV-2 infection may be subclinical in some subjects^[^^15^^]^. Genital herpes infection in pregnancy has been considered as a great concern, which was associated with a high risk of spontaneous abortions and intrauterine growth retardation^[^^15^^]^. 

Very limited surveys have evaluated the HSV-2 seropositivity status among pregnant women in Iran. In this regard, Rezaie-Chaparpordi *et al.*^[^^16^^]^ have reported a positive HSV-2 IgG antibody response in 28 (3.5%) subjects (in population from north of Iran). Also, another Iranian-based population has shown that the pooled prevalence of HSV infection in pregnant women was 0.64%^[^^17^^]^. Other survey, conducted in Tehran, Iran, has displayed that 8.25% of studied pregnant women are HSV-2 seropositive^[^^18^^]^. Since the HSV seropositivity is a potential sign of infection, it can be used to control the behavioral patterns for decreasing the HSV transmission. In this sense, the current commercial enzyme immunoassays with the ability to consistently distinguish between antibodies against HSV-1 and HSV-2 permit serological surveys to detect symptomatic- and asymptomatic-infected cases^[^^16^^]^. Thus, in this study, we aimed at evaluating the seroprevalence of HSV-2 in pregnant women referring to the public health centers in Urmia in northwest of Iran.

## MATERIALS AND METHODS

**Human subjects**

Subjects (n = 86) were randomly collected from pregnant women who were referred to health centers in Urmia (West Azerbaijan Province, Iran) for routine pregnancy follow-up and lived in different areas of the Urmia city. This study was carried out in September 2014 until May 2015 following the approval from the Scientific and Ethical Review Board of Urmia University of Medical Sciences (approval number: Ir.umsu.rec.1394.30). Before participating in the survey, all the subjects gave their informed consent for blood sampling, and HSV-2 serological assay was taken from each subject. In addition, each participant filled out a questionnaire containing demographic information, previous comorbidity, STDs history, blood transfusion, and any previous abortion or stillbirth. Demographic and clinical data were recorded on standardized forms. 

**Experimental protocols**

The blood samples (5 ml) were collected via venipuncture, and serum specimens were isolated and kept at -20 °C. For all the subjects, serologic tests were performed to detect HSV-2 seroprevalence by HSV-2-specific glycoprotein G2 using ELISA. Based on the protocol of the kit (Euroimmun Anti-HSV-2 [gG2] ELISA [IgG], Germany), the procedure and the interpretation of the results were completed.

**Statistical analysis**

Data were analyzed by SPSS statistical software (Version 16; Chicago, IL). Continuous variables were presented as mean and interquartile ranges, and categorical variables were shown as frequency and percentage. A Mann Whitney U test was used for comparing age between two HSV-2 seropositive and seronegative groups. Significant levels were considered statistically as *p* < 0.05.

## RESULTS

The mean ± SD age of patients was 25.5 ± 5.3 years, and 77 (89.5%) out of 86 inhabited in urban areas at the time of study, and the remnant (10.5%) lived in rural areas. In terms of education status, 30 subjects (34.8%) were illiterate, 52 (60.4%) studied until high school or below, and 4 (4.6%) had a diploma or a higher degree.

According to the serologic test, only 5 (5.8%) subjects were seropositive for anti-HSV-2 IgG. The patients’ characteristics of two HSV-2 seropositive and seronegative groups are presented in Table 1.

The mean age in the seropositive group was not statistically different from the seronegative group (*p* = 0.76). Most of the seropositive cases were from urban areas, with low educational level, without any history of previous comorbidity and blood transfusion. Only one subject in seropositive group had former STD infection, while 29 subjects (35.8%) in the seronegative group experienced at least one former example of STD infection. The age of starting sexual life (the mean values) was not significantly different between the two groups (*p* = 0.79). Concerning the number of marriages and partners of the participants, only one subject in the HSV-2 seronegative group reported to have more than one partner in her life (married twice). Previous abortion was reported in 16 (19.7%) seronegative and 2 seropositive subjects. Stillbirth was reported only in one case that was seronegative for anti-HSV-2 IgG.

## DISCUSSION

In the present survey, serum samples from 86 pregnant women were evaluated to detect the seropositivity of HSV-2. Based on our results, 5 (5.8%) of pregnant women had positive test results. In further analysis, the mean age, and age of marriage were not statistically different between HSV-2 positive and negative groups. One subject (20%) in the seropositive group and 29 (35.8%) in the seronegative group reported previous STD infection. 

**Table 1:** Comparison of the characteristics between seronegative and seropositive groups for anti-HSV-2 IgG

**Characteristics**	**HSV-2 IgG Seropositive** ** (n = 5)**			**HSV-2 IgG Seronegative ** **(n = 81)**
		
Age Median (IQR)	24 (8)			25 (10)
		
Place of birth Urban Rural				
	4 1			38 43
		
Residency Urban Rural				
	5 0			72 9
		
Education Illiterate Primary-high school Diploma/ higher				
	3 2 0			27 50 4
		
Age of 1^st^ pregnancy Median (IQR)		18 (8)		19 (4)
Primipara Multipara	1 4			33 48
		
Gestational Age 1^st^ semester 2^nd^ semester 3^rd^ semester				
	0 3 2			23 29 29
		
Previous comorbidity (%)	0			2 (2.4)
History of blood transfusion (%)		0		3 (3.7)
History of STD infection (%)			1 (20)	29 (35.8)
History of abortion (%)	2 (40)			16 (19.7)

IQR, interquartile range

Based on a previous study, HSV-2 is the main cause of genital ulcer in the developing world^[^^19^^]^. In this regard, in a similar study, which has been carried out in Tehran, Iran, the prevalence of IgG antibody in high-risk women was significantly higher than low-risk women (26.3% vs. 2.5%)^[20]^. In another survey, HSV-2 seropositivity was assessed in pregnant women at labor stage in Tehran, Iran; the results showed that 33 (8.25%) of tested pregnant subjects were seropositive for HSV-2 antibodies^[^^18^^]^. The rate of HSV-2 positive cases in these studies was higher than our results, which could be related to cultural differences in these areas. 

A very recent survey, which evaluated the prevalence of HSV-2 IgG among sheltered homeless adults in Tehran, Iran, has shown that the HSV-2 prevalence in this group was more than the general population. The high rate of HSV-2 prevalence in sheltered homeless adults might be related to the fact that infectious diseases are more prevalent between homeless people. Indeed, their living status and weak sanitation are the factors for high risks of infectious diseases among them, which leads to higher susceptibility to numerous infections^[^^21^^]^. A study on the seroepidemiology of HSV-2 in HIV-infected subjects in Kermanshah, Iran has demonstrated that among 170 cases, 11 (6.5%) were seropositive for HSV-2. In HIV-infected subjects, seroprevalences in female and male were 17.6% and 5.2%, respectively^[^^22^^]^.

According to the fact that HSV-2 infection has been frequently reported to be growing in women^[^^22^^]^, we indicated that its lower rate in our survey may be due to the small number of participants. Also, a former report from England and Wales identified HSV-2 antibody in sera from 3.3% of men and 5.1% of women^[^^23^^]^. Another study from Turkey indicated that the total IgG seropositivity rates for HSV-2 was 8.2% in asymptomatic pregnant women^[^^24^^]^. Based on our findings, there were no significant differences between seronegative and seropositive groups for any of the following features: age at the first sexual contact and the number of sexual partners. The seropositive cases were more from the low educational level, but since we had very few seropositive cases, we could not make any conclusion in this regard. In Kim *et al.*'s^[^^2^^]^ study, the rate of spontaneous abortion was higher in HSV-2 seropositive women than healthy controls though the trans-placental passage of the virus was rare.

In our survey, previous abortions were reported in 16 (19.7%) subjects of the seronegative group and 2 (40%) subjects of the seropositive group. However, due to the small number of seropositive cases, no conclusion could be made here. Development of serologic diagnostic methods, leading to sero-epidemiological studies, has suggested an increase in the prevalence rate of HSV-2-related genital herpes in most countries^[^^23^^,^^24^^]^. In this study, we did not evaluate the subjects for the presence of anti-HSV-2 IgM; hence, regarding the presence of HSV-2 infection during pregnancy in our population, it was impossible to deduce any conclusion. Since in this survey all subjects were chosen randomly amongst pregnant women who were referred to Urmia public health centers (neglecting referrals to the private centers); therefore, absence of pregnant subjects with higher socio-economic or educational levels might be assumed as one of the study limitation. 

 In conclusion, the seroprevalence of HSV-2 among our studied pregnant women from Urmia, Northwest of Iran, was not noticeable in comparison to other surveys. However, the number of evaluated samples was low, and it seemed that these samples were not sufficient for a definitive and certain statement. Indeed, the results of the present study could be considered as a primary data, and further experiments with the higher number of samples are needed to reach the certain conclusion.

## CONFLICT OF INTEREST.

None declared. 
